# Machine Perfusion Across Marginal Liver Grafts: Benefits and Challenges

**DOI:** 10.3390/jpm16040228

**Published:** 2026-04-20

**Authors:** Leandro Sierra, Maria Ortega Abad, Maria Saavedra-Martinez, Kanisha Bahierathan, Zainab Ifthikar, Ana Eliza Velez, Nikki Duong, Luis Antonio Diaz, Juan Pablo Arab

**Affiliations:** 1 Department of Internal Medicine, Cleveland Clinic, Cleveland, OH 44195, USA; ifthikz@ccf.org; 2 Endocrinology and Metabolism Institute, Cleveland Clinic, Cleveland, OH 44195, USA; ortegam3@ccf.org; 3 Facultad de Medicina, Universidad de La Sabana, Chía 250001, Colombia; mariasaama@unisabana.edu.co; 4 School of Medicine, Case Western Reserve University, Cleveland, OH 44195, USA; ksb128@case.edu; 5 Facultad de Medicina, Universidad del Azuay, Cuenca 010105, Ecuador; anaeli.vf@hotmail.com; 6 Division of Gastroenterology and Hepatology, Stanford University, Palo Alto, CA 94304, USA; nduong91@stanford.edu; 7 MASLD Research Center, Division of Gastroenterology and Hepatology, University of California San Diego, San Diego, CA 92093, USA; luisdiazpiga@gmail.com; 8 Departamento de Gastroenterología, Escuela de Medicina, Pontificia Universidad Católica de Chile, Santiago 8331150, Chile; jparab@gmail.com; 9 Division of Gastroenterology, Hepatology, and Nutrition, Department of Internal Medicine, School of Medicine, Virginia Commonwealth University, Richmond, VA 23298, USA

**Keywords:** machine perfusion, liver transplantation, marginal grafts, extended criteria donors, organ preservation

## Abstract

Liver transplantation is the definitive therapy for end-stage liver disease, yet persistent organ shortages result in approximately 10% of recovered livers being discarded, with markedly higher discard rates among marginal grafts from elderly donors, donation after circulatory death (DCD), and those with macrovesicular steatosis. Machine perfusion (MP) has emerged as a paradigm-shifting preservation strategy with the potential to safely expand the usable donor pool. This narrative review examines the current evidence for three MP modalities—hypothermic machine perfusion (HMP), normothermic machine perfusion (NMP), and normothermic regional perfusion (NRP)—across various marginal donor populations, including elderly donors, steatotic grafts, donors with infectious diseases, and split liver transplantation. Current evidence demonstrates that MP significantly increases utilization of steatotic grafts with up to an eightfold rise in usage of severely steatotic organs. HMP consistently reduces non-anastomotic biliary strictures and early allograft dysfunction across donor types, while NMP enables real-time viability assessment and reduces post-reperfusion syndrome in steatotic grafts. NRP shows particular benefit in DCD organs, reducing biliary complications and improving one-year survival. Additionally, MP extends preservation times enabling next-day split liver transplantation and shows promise as a platform for ex situ antiviral therapy. Despite compelling evidence supporting MP in marginal grafts, widespread adoption remains constrained by high costs, logistical complexity, and the absence of standardized protocols. Future progress will require multicenter studies evaluating long-term outcomes alongside consensus-driven implementation frameworks.

## 1. Introduction

Liver transplantation (LT) is the definitive therapy for end-stage liver disease, yet a persistent mismatch between organ supply and demand continues to limit access to life-saving care. Approximately 10% of recovered livers remain unused, with markedly higher discard rates among grafts from older donors, donation after circulatory death (DCD), and macrovesicular steatosis, prompting transplant centers to explore strategies to safely expand the usable donor pool [1]. Marginal livers now represent viable opportunities for donor pool expansion. Livers from elder donors, donors with human immunodeficiency virus (HIV) and steatosis have shown acceptable outcomes when carefully selected [2,3,4]. Nevertheless, utilization remains inconsistent across transplant centers [5].

Machine perfusion (MP) has emerged as a paradigm-shifting organ preservation strategy with the potential to optimize outcomes in marginal liver allografts. For more than three decades, static cold storage (SCS) served as the standard method of organ preservation, relying on hypothermic flushing with preservation solution and transport on ice at approximately 4 °C. Although SCS is simple and cost-effective, prolonged cold ischemia leads to mitochondrial dysfunction, depletion of adenosine triphosphate (ATP), and accumulation of harmful metabolites [6]. By restoring cellular energy stores, improving mitochondrial function, and attenuating ischemia–reperfusion injury, MP has transformed the landscape of LT and has been associated with measurable reductions in organ nonuse [7,8]. These benefits are especially important in extended criteria donors (ECD), where the physiological advantages of MP may outweigh the graft failure risks.

Despite its promise, the widespread adoption of MP remains limited by high costs, lack of standardized utilization criteria, and a paucity of long-term outcome data [9]. Accordingly, this review aims to critically examine the benefits and challenges of MP across donor populations, with a focus on its role in expanding the donor pool and improving transplant outcomes.

## 2. Overview of Machine Perfusion Modalities

Liver MP represents an advanced strategy for donor liver preservation, involving the circulation of an oxygenated solution through the organ’s vascular network, either ex situ or in situ. MP systems integrate oxygenators, temperature regulators, and nutrient reservoirs, with pumps ensuring controlled perfusate flow throughout the liver. These techniques are broadly classified according to the perfusion temperature. Hypothermic protocols aim to reduce cellular metabolism and preserve mitochondrial function, whereas normothermic protocols maintain the organ at physiological temperatures, enabling real-time assessment of graft viability [8,10].

**Figure 1:**
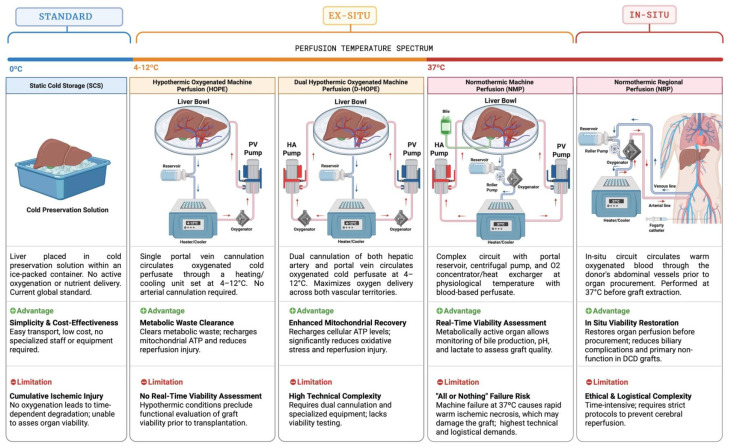
Comparison of liver preservation modalities across the perfusion temperature spectrum.

Three MP modalities have been described: hypothermic machine perfusion (HMP), normothermic machine perfusion (NMP), and normothermic regional perfusion (NRP). HMP employs perfusate at 4–12 °C and is further subdivided into portal vein–only oxygenated perfusion (HOPE) and dual perfusion of portal vein and hepatic artery (D-HOPE) (Figure 1) [10]. Clinical studies have associated HOPE with reductions in early allograft dysfunction, 12-month graft failure, re-transplantation, and overall biliary complications [10,11,12]. However, the hypothermic nature of HMP limits real-time functional assessment, as deviation from physiological temperature precludes comprehensive evaluation of graft viability before transplantation [10].

Static cold storage (SCS) at 0 °C is the current global standard providing no active oxygenation. Ex situ hypothermic oxygenated machine perfusion (HOPE, 4–12 °C) uses single portal vein cannulation to circulate oxygenated cold perfusate, clearing metabolic waste and recharging mitochondrial ATP stores. Dual hypothermic oxygenated machine perfusion (D-HOPE) adds hepatic artery cannulation to maximize oxygen delivery across both vascular territories. Normothermic machine perfusion (NMP, 37 °C) employs a blood-based perfusate at physiological temperature, enabling real-time viability assessment through bile production, pH, and lactate monitoring. Normothermic regional perfusion (NRP) is an in situ technique that restores warm oxygenated blood flow through the donor’s abdominal vessels at 37 °C prior to organ procurement, reducing biliary complications and primary non-function. Arrows indicate the direction of perfusate flow through the vascular circuit. HA, hepatic artery; PV, portal vein.

NMP utilizes oxygenated hemic solutions at 35–38 °C, closely mimicking in vivo conditions [8]. By sustaining near-physiological temperatures, NMP allows comprehensive pre-transplant functional assessment, including real-time monitoring of lactate clearance, biliary output, and glucose homeostasis. These features are associated with decreased ischemia–reperfusion injury, lower incidence of early allograft dysfunction, and increased salvage of DCD grafts [7,8].

NRP is an in situ strategy for DCD organs, employing a circuit to circulate warm, oxygenated blood through the donor prior to procurement. Abdominal NRP (A-NRP) targets abdominal organs, whereas thoraco-abdominal NRP (TA-NRP) is used when thoracic and abdominal organs are procured. This approach facilitates in situ viability assessment and reduces graft dysfunction and biliary complications. However, NRP is time-intensive and raises ethical considerations regarding the definition of death, necessitating strict protocols to prevent cerebral reperfusion and ensure proper death confirmation [13].

## 3. Machine Perfusion Across Marginal Liver Grafts

Marginal liver grafts are historically associated with poorer post-LT outcomes, which have limited their utilization. This category primarily includes donors with characteristics that increase the risk of graft failure, disease transmission, or donor-related complications [14,15]. ECD are a key subset and encompass donors with advanced age, significant macrovesicular steatosis, donation from cardiovascular death (DCD), organ dysfunction at procurement, death from anoxia or cerebrovascular accident, infectious diseases (hepatitis B virus [HBV], hepatitis C virus [HCV], HIV), extrahepatic malignancy, or prolonged cold ischemia time (>12 h) [3,16].

MP provides established benefits across this category of donors; however, these vary by perfusion modality. A meta-analysis has shown that HMP provides the most consistent benefits, including lower rates of non-anastomotic biliary strictures and early allograft dysfunction, as well as improved 1-year survival compared with SCS across donation after brain death (DBD), DCD, and ECD [17]. Complementing these findings, a recent network meta-analysis of 12 randomized controlled trials including 1628 patients further characterized the comparative performance of individual MP modalities against SCS. Among all strategies analyzed, HOPE and dual-HOPE were the only modalities associated with statistically significant improvements across multiple endpoints, including reduced early allograft dysfunction (RR: 0.53; 95% CI [0.37–0.74]), higher one-year graft survival (RR: 1.07), lower graft failure rates (RR: 0.38), and fewer biliary complications (RR: 0.52). NMP demonstrated a significant reduction in post-reperfusion syndrome but did not confer additional benefit over SCS in other outcomes. No MP strategy was associated with differences in patient mortality, need for renal replacement therapy, or length of hospital or ICU stay, suggesting that the clinical advantage of MP at this stage remains primarily graft-centered [18]. A recent Polish randomized control trial found no advantage of HMP in DBD grafts with a donor risk index (DRI) < 1.7, whereas a significant improvement in 2-year graft survival was observed among those with DRI > 1.7 (100% with HMP vs. 73.1% with SCS), suggesting that HMP might need to be reserved for high DRI grafts [19].

On the other hand, meta-analytic data on NMP demonstrated that it reduces major complications in ECD and early allograft dysfunction in DCD yet had no effect in survival across donor types. However, the 2022 International Liver Transplantation Society consensus recommends NMP use for marginal grafts and the VITTAL clinical trial demonstrated that NMP enables successful transplantation of livers from ECD with 100% 90-day patient and graft survival [20]. A multicenter RCT across 15 U.S. transplant centers randomizing 383 donor livers to NMP versus SCS found a numerically lower rate of early allograft dysfunction with NMP (20.6% vs. 23.7%); while the primary endpoint did not reach statistical significance, post hoc analyses suggested greater benefit among higher-risk donor livers, supporting the selective use of NMP in marginal grafts [21]. NRP showed significant benefit among DCD only, with reduced non-anastomotic biliary strictures, early allograft survival, primary non-function and hepatic artery thrombosis, as well as improvements in 1-year survival, highlighting its benefit as a graft preservation technique, particularly among countries with long non-touch times after DCD [22,23]. The most relevant clinical studies evaluating MP among specific marginal graft types are described in Table 1 [10,11,12,22,23,24,25,26,27,28,29,30,31].

### 3.1. Elderly Donors

MP has shown promising results in elderly donors. In a cohort of 844 liver transplants, D-HOPE was associated with reduced early allograft failure (OR 0.24, *p* = 0.024), fewer grade ≥ 3 complications (OR 0.57, *p* = 0.046), and improved 90-day and 1-year graft survival, with effects most pronounced in elderly donors [32]. In contrast, NMP appears to confer histological benefits but less consistent clinical improvement. A randomized trial of 20 liver grafts from donors aged ≥70 years found that NMP, compared to CSC, attenuated ischemia–reperfusion injury, as evidenced by reduced mitochondrial volume density and increased autophagy; however, peak transaminases were similar between groups. U.S. data further suggest that ex vivo NMP may benefit marginal grafts from donors > 60 years by reducing early allograft dysfunction and facilitating organ utilization [33]. A meta-analysis of 690 livers undergoing NMP for viability assessment reported utilization rates of 82% in DBD and 68% in DCD donors, with donor age and warm ischemia time not independently predicting the transplant decision, further supporting NMP as an objective assessment tool across varying donor risk profiles [34].

### 3.2. Steatotic Grafts

MP has significantly increased the utilization of macrosteatotic grafts, with nearly an eightfold rise in the use of grafts with >30% macrosteatosis (OR 7.89, 95% CI 3.76–16.58; *p* < 0.001), with no detrimental effect on 1- and 3-year graft survival [29]. Compared to SCS, ex situ NMP significantly reduces post-reperfusion syndrome (9.5% vs. 37.5%; *p* < 0.001), and early allograft dysfunction (42.9% vs. 76.4%; *p* < 0.001) in grafts with moderate (30–60%) macrosteatosis, translating into improved patient and graft survival [30]. Emerging pharmacological defatting strategies during MP are a promising frontier for reconditioning steatotic grafts. These interventions target hepatic lipid metabolism pathways to reduce triacylglycerol accumulation and improve graft viability, potentially expanding the pool of transplantable steatotic livers beyond what perfusion alone can achieve [35,36].

### 3.3. Donors with Infectious Diseases

Across donors with infectious diseases, MP has been evaluated mainly in HCV-positive grafts as a platform for ex situ antiviral therapy. Preclinical studies show enhanced antiviral drug uptake and greater HCV suppression with NMP compared with SCS, highlighting the potential to reduce post-transplant HCV recurrence [37]. To date, no studies have specifically evaluated MP in HBV or HIV-positive grafts. A multicenter U.S. study found no differences in 1-year graft survival, rejection, or HIV breakthrough between HIV-positive and HIV-negative donors transplanted into HIV-positive recipients; however, preservation was performed exclusively with static cold storage (SCS) [3]. It is plausible that the benefits of MP may extend to this population, as they do with other marginal grafts, with potential improvements in survival and disease recurrence.

### 3.4. Split Grafts

A major challenge with split LT has been finding two recipients on the same day, as a consequence, one liver graft risks not being used in these cases. MP has the potential to prolong preservation time, enabling safe second split-graft transplantation the following day. In a series of 6 adult split liver grafts, NMP preservation allowed transplantation on the second day with a mean perfusion time of 17 h (±5.2 h), all grafts met viability criteria, and 90-day graft and patient survival was 100% [38]. 

Likewise, HMP has shown significant benefits in split LT. In a porcine model comparing 6 h SCS plus 2 h HOPE versus 8 h SCS alone, HOPE demonstrated improvements in biochemical markers (AST and LDH) immediately after reperfusion, and significantly lower inflammatory cytokine levels (TNF-α, IFN-γ, IL-1β, and IL-10) [39]. Clinical data further support these findings. D-HOPE during full-left-full-right split LT for two adult recipients demonstrated excellent one-year outcomes, with stable graft function and no major complications [40]. Furthermore, HOPE provides a novel approach to split LT by performing the actual splitting procedure during HOPE (HOPE-Split). In 16 consecutive HOPE-Split transplants (8 procedures creating grafts for both adult and pediatric recipients), all grafts were successfully transplanted with no graft loss or recipient death at median 7.5-month follow-up [31].

While each modality offers distinct mechanistic advantages, the clinical evidence points to meaningful differences in their respective roles. HOPE and dual-HOPE provide the most consistent graft-level benefits across donor types, particularly in reducing early allograft dysfunction and biliary complications, and represent the most reproducible strategy for high-risk DCD and elderly donors. NMP, despite greater logistical demands, remains the only modality enabling real-time viability assessment, which is especially relevant when the transplant decision itself is uncertain, as in borderline steatotic grafts. NRP is best suited to controlled DCD settings where prolonged non-touch times are standard, offering meaningful reductions in ischemic cholangiopathy that the ex situ strategies do not replicate. Rather than competing approaches, these modalities are better understood as complementary tools whose selection should be driven by donor phenotype, institutional capacity, and the clinical objective at hand [10,17,18,20,22,23].

## 4. Practical Challenges and Implementation Barriers

Despite growing evidence that MP enhances utilization of marginal grafts and improves recipient outcomes, it is not used routinely due to various challenges and implementation barriers. Navigating the costs for MP being a primary issue, the question of who should assume responsibility for offsetting these substantial costs on behalf of patients remains unresolved [41].

Key expenditures associated with MP include disposable perfusion circuits and machine components, as well as the need for highly trained personnel to provide continuous monitoring, organ transportation, and storage. Collectively, these factors can raise the cost of a single transplant by more than $18,000 [42]. A single-center comparison of 144 NMP and 149 SCS cases confirmed significantly higher index hospitalization costs with NMP ($256,810 vs. $209,144), driven primarily by organ acquisition costs ($135,930 vs. $50,940); however, NMP was associated with shorter operative times, reduced ICU stay, and a markedly higher rate of daytime case starts (88.9% vs. 46.3%), highlighting the complex cost–benefit trade-offs that programs must consider before implementing NMP [43]. NMP has also demonstrated a measurable impact on transplant workflow. Using national UNOS data, Wang et al. demonstrated that NMP significantly increased the proportion of daytime transplants, with potential benefits for operating room scheduling, staff efficiency, and overall program logistics [44]. Beyond per-transplant costs, implementation of a routine NMP program at two academic centers was associated with a reduction in median waitlist time from 79 to 14 days and lower total healthcare costs during the waitlist period, suggesting that the broader systemic benefits of NMP may offset its upfront expenditures [45]. For smaller healthcare and transplant centers, the upfront investment required for such advanced technology may be prohibitive, placing them at a competitive disadvantage compared with larger, better-resourced institutions [9,41]. However, emerging data suggest that the initially high per-transplant cost may be justified by downstream benefits, including reduced postoperative complications and lower cumulative healthcare expenditures within the first year after transplantation [46]. A single-center analysis demonstrated that despite higher hospitalization costs, NMP significantly reduced operative time, ICU and hospital length of stay, and facilitated the transition of cases to daytime hours [43].

Complex transportation of machine-perfused livers presents additional logistical challenges that may hinder widespread adoption. Unlike SCS, MP requires the organ to remain connected to a functioning device throughout transit, necessitating reliable power sources, secure positioning of sensitive equipment, and trained personnel capable of monitoring perfusion parameters in real time. This process is multi-layered, often requiring specialized transport arrangements and meticulous coordination between procurement teams, transport services, and the recipient center. Delays, technical malfunctions, or limited device availability can disrupt transplant scheduling and potentially create bottlenecks in organ allocation and operating room workflows and introduce system-level constraints [9]. Furthermore, NMP has been shown to shift transplant caseloads from nighttime to daytime hours, potentially improving workflow efficiency [44].

Another significant logistical and clinical barrier to the widespread implementation of liver MP is the absence of sufficiently standardized protocols governing perfusion timing and graft selection. At present, there is no universal consensus regarding the optimal duration of perfusion or the most appropriate temperature strategy, whether normothermic or hypothermic, resulting in substantial inter-center variability. Programs differ in how long livers are perfused, which biochemical and functional parameters are emphasized, and how these metrics are interpreted in real time [47].

Similarly, clear and consistently applied guidelines defining which donor livers derive the greatest benefit from MP remain lacking. Uncertainty persists as to whether perfusion should be selectively reserved for ECD or broadly applied to all allografts. Moreover, debate continues over whether organs at the highest risk of early allograft dysfunction should be preferentially prioritized for perfusion or whether such risk stratification may inadvertently introduce allocation bias. Discrepancies in viability assessment thresholds further compound this variability, as centers rely on differing combinations of lactate clearance, bile production, hemodynamic stability, and transaminase trends to guide organ acceptance [48,49]. 

This heterogeneity not only creates uncertainty in clinical decision-making but also limits reproducibility, outcome comparability, and scalability across transplant programs. For liver MP to achieve widespread and equitable adoption, the LT community must collaborate to establish evidence-based, consensus-driven guidelines that standardize perfusion protocols and graft selection criteria.

## 5. Future Directions

Although compelling evidence supports MP in DCD, elderly, steatotic, and split grafts, its role in other marginal donor populations remains insufficiently defined, particularly among donors with chronic viral infections (HCV, HBV, HIV) and prior malignancy. Future investigations should prioritize these understudied groups and incorporate head-to-head evaluations of perfusion strategies within biologically defined donor phenotypes, recognizing that distinct technologies may confer differential benefit depending on graft characteristics. Concurrently, robust health economic and cost-effectiveness analyses are required to delineate the long-term value of MP and to inform policy decisions that support equitable implementation.

## 6. Conclusions

MP is redefining liver preservation by enabling safer utilization of marginal grafts. Across multiple donor populations, perfusion strategies reduce ischemia–reperfusion injury, support viability assessment, and increase graft utilization, with the greatest benefits observed in marginal organs. However, widespread adoption remains constrained by cost, logistical complexity, and the lack of standardized protocols for graft selection and viability criteria. Future progress will depend on multicenter data clarifying long-term outcomes and cost-effectiveness, alongside consensus-driven implementation frameworks. As technologies evolve, MP is poised to serve not only as a preservation tool but as a therapeutic platform, expanding the transplantable organ pool and improving access and outcomes in LT.

## Figures and Tables

**Table 1:** Clinical Evidence for Machine Perfusion Strategies in Specific Types of Marginal Donor Liver Transplantation.

Study	Design	Machine Perfusion Type	Donor Age, yr ^1^	Donor Risk Index ^1^	Donor Type	*n* (MP)	Early Allograft Dysfunction (%)	Biliary Complications (%)	90-Day Graft Survival (%)	1-Year Graft Survival (%)
Donation After Circulatory Death										
Dutkowski et al., 2015 [12]	Retrospective	HOPE	54 (36–63)	NR	DCD (Maastricht III)	25	20 vs. 44 *	20 vs. 46 *	NR	90 vs. 69 *
van Rijn et al., 2017 [24]	Prospective	D-HOPE	53 (47–57)	1.9 (1.47–2.19)	DCD (Maastricht III)	10	NR	40 vs. 65 *	NR	100 vs. 67
Schlegel et al., 2019 [11]	Retrospective	HOPE	57 (47–67)	NR	DCD	50	NR	IC: 0 vs. 10 * AS: 24 vs. 18 BL: 2 vs. 2	NR	95 vs. 81
Watson et al., 2019 [25]	Retrospective	NRP	41 (33–57)	1.8 (1.7–2.4)	cDCD (Maastricht III)	43	12 vs. 32 *	IC: 0 vs. 27 * AS: 7 vs. 27 * BL: 7 vs. 10	100 vs. 97	NR
van Rijn et al., 2021 [10]	RCT	D-HOPE	52 (43–57)	2.12 (1.84–2.38)	DCD (Maastricht III)	78	26 vs. 40 *	AS: 29 vs. 28 NAS: 6 vs. 18 * BL: 8 vs. 10	NR	NR
Hessheimer et al., 2022 [23]	Retrospective	NRP	56 (49–67)	NR	cDCD (Maastricht III)	95	22 vs. 27 (SRR)	8 vs. 31 (SRR) *	NR	88 vs. 83 (SRR)
Brubaker et al., 2024 [22]	Retrospective	NRP	30.5 (22–44)	NR	cDCD (Maastricht III)	106	36.4 vs. 56.1 (SRR) *	IC: 0 vs. 9 (SRR) * AS: 6.7 vs. 22.4 (SRR) *	NR	98 vs. 88 (SRR) ^e^
Elderly Donors										
Ghinolfi et al., 2019 [26]	RCT	NMP	81 (77.5–87.2)	NR	DBD ≥ 70 yr	10	20 vs. 10	10 vs. 0	NR	NR
Patrono et al., 2022 [28]	Retrospective	D-HOPE	82.7 (80.1–83.7)	2.4 (2.2–2.5)	DBD ≥ 75 yr	63	0 vs. 8.5	IC: 4.8 vs. 5.6 AS: 20.6 vs. 15.8	NR	98 vs. 88 ^e^
Torri et al., 2024 † [27]	RCT sub-analysis	D-HOPE vs. NMP	80 (77–85)	NR	cDCD ≥ 70 yr	D-HOPE: 6 NMP: 5	17 vs. 20 (NMP)	0 vs. 20 (NMP)	NR	NR
Steatotic Grafts										
Cywes et al., 2024 [29]	Retrospective	MP unspecified type	49.5 (36.5–59.0)	NR	MaS ≥ 30%	72	NR	NR	NR	89 vs. 87.5 (No MP)
Zhang et al., 2025 [30]	Retrospective cohort	NMP	52.3 ± 12.6	NR	Moderate MaS (30–60%)	63	42.9 vs. 76.4 *	IC: 1.6 vs. 3.1 AS: 20.6 vs. 12.5	NR	100 vs. 91.6
Split Grafts										
Rossignol et al., 2022 [31]	Prospective pilot	HOPE	21 (19–27)	2.04 (1.88–2.23)	Split grafts	8	25 vs. 58	12 vs. 16	NR	NR

^1^ Donor age and Donor Risk Index (DRI) are expressed as mean with interquartile range (IQR) unless otherwise specified. Static cold storage (SCS) served as the comparator in all studies unless an alternative reference is explicitly indicated. Biliary complications represent overall biliary complication rates unless a specific subtype is designated [anastomotic stricture (AS), non-anastomotic stricture (NAS), ischemic cholangiopathy (IC), or bile leak (BL)]. ^e^ Estimated graft survival based on Kaplan–Meier analysis as reported in the original publication. † Comparator is normothermic machine perfusion (NMP), not SCS. * Statistically significant difference versus comparator (*p* < 0.05). Abbreviations: AS, anastomotic stricture; BL, bile leak; cDCD, controlled donation after circulatory death; D-HOPE, dual hypothermic oxygenated machine perfusion; DCD, donation after circulatory death; DBD, donation after brain death; HOPE, hypothermic oxygenated machine perfusion; IC, ischemic cholangiopathy; IQR, interquartile range; MaS, macrovesicular steatosis; MP, machine perfusion; NAS, non-anastomotic stricture; NMP, normothermic machine perfusion; NR, not reported; NRP, normothermic regional perfusion; RCT, randomized controlled trial; SCS, static cold storage; SRR, super-rapid recovery; yr, year.

## Data Availability

No new data were created or analyzed in this study. Data sharing is not applicable to this article.

## Abbreviations

The following abbreviations are used in this manuscript:

A-NRP	Abdominal normothermic regional perfusion
AS	Anastomotic stricture
ATP	Adenosine triphosphate
BL	Bile leak
cDCD	Controlled donation after circulatory death
D-HOPE	Dual hypothermic oxygenated machine perfusion
DBD	Donation after brain death
DCD	Donation after circulatory death
DRI	Donor risk index
EAD	Early allograft dysfunction
ECD	Extended criteria donors
GS	Graft survival
HA	Hepatic artery
HBV	Hepatitis B virus
HCV	Hepatitis C virus
HIV	Human immunodeficiency virus
HMP	Hypothermic machine perfusion
HOPE	Hypothermic oxygenated machine perfusion
IC	Ischemic cholangiopathy
IQR	Interquartile range
LT	Liver transplantation
MaS	Macrovesicular steatosis
MP	Machine perfusion
NAS	Non-anastomotic stricture
NMP	Normothermic machine perfusion
NR	Not reported
NRP	Normothermic regional perfusion
PV	Portal vein
RCT	Randomized controlled trial
SCS	Static cold storage
SRR	Super-rapid recovery
TA-NRP	Thoraco-abdominal normothermic regional perfusion
